# Effect of task nature during short digital deprivation on time perception and psychophysiological state

**DOI:** 10.1038/s41598-025-94316-3

**Published:** 2025-03-26

**Authors:** Quentin Meteier, Anouk Délèze, Sébastien Chappuis, Joanna Witowska, Marc Wittmann, Ruth Ogden, Chantal Martin-Sölch

**Affiliations:** 1 Clinical and Health Psychology Unit, Department of Psychology, University of Fribourg, Fribourg, 1700 Switzerland; 2 Institute of Psychology, The Maria Grzegorzewska University, Warsaw, 02-353 Poland; 3 Institute for Frontier Areas of Psychology and Mental Health, 79098 Freiburg, Germany; 4 School of Psychology, Liverpool John Moores University, Liverpool, L3 3AF England

**Keywords:** Psychology, Human behaviour

## Abstract

The technological advances in recent years are influencing and redefining our daily lives, communications, and social relationships. While these advances bring us many benefits, their negative effects may also cause concern. Although often studied, the potential benefits of digital deprivation are still disputed. This laboratory study investigates the impact of short digital deprivation (7 min and 30 s) on the psychophysiological state and time perception of 90 participants. Three experimental conditions were created for the task performed during the waiting period (30 subjects per condition). Participants had to either freely use their smartphone, perform a non-digital task (sudoku), or wait (i.e. passive digital deprivation). Indicators of electrodermal activity and heart rate variability were calculated for the baseline and waiting periods, along with measures of subjective affective state. Four measures of time perception were also collected after the waiting period. Regardless of their experimental condition, the participants underestimated the duration of the waiting period on average (5 min 44 vs. 7 min 30). Passive digitally deprived participants felt that the time passed more slowly and were more bored than participants engaged in a task, regardless of whether the task was digital or not. Sudoku induced more positive affect and was more cognitively engaging than the free use of a smartphone regarding heart rate variability measures. The results suggest that performing a digital task (free smartphone use) is less cognitively demanding than a non-digital task (sudoku) and alters time perception in the same way. The digital nature of a task might also impact one’s affective reaction. A similar study in the field with longer or repetitive digital deprivation periods and a different non-digital task to perform (e.g., reading news) should be conducted to confirm the results obtained in this study.

## Introduction

In recent years, the digitalization of modern society has accelerated, revolutionizing many aspects of daily life. In particular, the COVID-19 pandemic has greatly intensified the need for digital transformation, forcing companies and individuals to react quickly to change and develop solutions^1^. Technology is no longer simply an accessory tool but has become an essential element around which today’s society is built^2^. These technological advances influence and redefine our daily lives, from the way we work, communicate and interact with others, to the way we think and perceive the world^3–5^.

It is clear that digitalization brings considerable benefits such as enhanced sustainability, improved productivity and economic growth, or increased social connectedness^6–9^, for young and older adults^10,11^. However, its negative effects are a growing concern^4^. Recently, many studies have expressed issues about technology’s increasing use and ubiquity. These concerns are based on a wealth of research arguing that the use of digital tools has considerable negative impacts on physical and mental health^4,12–15^, on the quality of social interactions^4,16,17^, on academic abilities^13^ as well as on our emotional state^4,13,14,18,19^.

To mitigate these negative effects, several studies have investigated the concept of “digital detox”^20–22^. Its effects on psychological and physiological well-being are regularly investigated but remain unclear^21–26^. A pilot study on smartphone usage found decreased levels of stress after a digital detox week^23^, but another randomized experimental study on social media use showed that complete abstinence resulted in increased negative affect and loneliness, with less life satisfaction^24^. Mixed results were also found in another study focusing on social media use in similar conditions^26^, and in a systematic literature review investigating the effect of digital detox interventions on health, wellbeing, social relationships, self-control or performance^21^.

Phone deprivation can also emotionally affect its user, be partially mediated by fear of missing out (FOMO) and increased levels of anxiety^27^. The inability of participants to answer their ringing phone also lead to increased feelings of anxiety, unpleasantness, increased heart rate (HR) and blood pressure among others^28^.

Another perhaps more subtle aspect on which digitalization can also have an impact is time perception. Regular immersion in the virtual world can affect temporal perception, in which minutes are subjectively prolonged or lost^29,30^. Although studies have been carried out, the link between digitalization and time perception remains complex and still needs clarification. For instance, some studies suggest playing video games induces a loss of time perception due to emotional factors experienced in states of flow^31^, while other studies indicate improvements in temporal discrimination and cognition^29,32–34^. Besides, other external factors such as the context in which we find ourselves, or internal factors such as impulsivity trait or body temperature, influence time perception^35–37^.

While some studies have been conducted to understand the effect of digitalization on time perception, none have been carried out on the effect of digital deprivation on both psychophysiological state and time perception. Furthermore, the effect of digital deprivation on mental and physical health has yielded inconsistent results, partly due to variations in experimental design^21^. Therefore, the present study addresses these issues by examining the effects of short digital deprivation on time perception and psychophysiological state together. We formulate the following research questions : (1) *“Do digitally deprived people perceive the passage of time differently than non-deprived people while waiting?”*, (2) *“When engaged in a task while waiting*,* does the digital nature of a task have an influence on time perception?”* and (3) *“Are the effects on psychophysiological measures correlate with time perception measures?”*.

## Literature review

### Digitalization and digital deprivation

Digitalization is characterized by the initiation or expansion of the use of digital tools by individuals, companies, and organizations (e.g., in education)^38^. These digital tools encompass the fields of communication, information, computer science, and technology^39^. Specifically, they can be divided into two categories: the first, referred to as primary, includes the smartphone, social networks, the Internet, and data storage and analysis, the second, more recent, covers artificial intelligence, augmented or virtual reality, 3D printers, accessories (such as watches), robots or algorithms^40^. Overall, digitalization can impair both our social and professional lives. Social networks and media are often mentioned in the literature, with ambivalent results as to their negative impact^4,9,10,41^. To illustrate some of the negative effects found, studies have pointed out their impact on social comparisons, FOMO or exposure to negative images and tragedies leading to increasing anxiety^42–44^. Problematic usage of internet and related concepts such as internet addiction have also been investigated as potential mental health concerns^45–47^. Negative effects or links found in prior studies include conduct problems, hyperactivity and physical health in children and adolescents^48^, social anxiety^49^ and clinical comorbidities such as mood, anxiety, impulse control and addictive disorders^46^ among others. Furthermore, the concept of technostress, which arises from the inability to adapt to new technology, is also an emerging issue^50^. Additional psychological negative effects are occasionally mentioned. For example, digital overexposure at work can lead to burnout^51^, and hyperconnectivity, which can disrupt the balance between life and work^52^.

To mitigate the negative effects that digitalization can have on our lives, previous research has investigated the benefits of not using electronical devices, referring to the term “digital detox”^21^. Digital detox interventions are defined as “timeouts from using electronic devices, such as smartphones, to reduce negative impacts from smartphone use on well-being or social relationships”^21^. They can range from several hours to several days^22,23^. Other terms are used to refer to similar interventions such as “social media abstinence”^24,26^, or “smartphone abstinence”^22^. When referring to the involuntary separation from smartphones, terms such as “cell phone separation”, “smartphone separation” or “phone deprivation” are employed in the literature^27,28^. Given the heterogeneity of the definitions and concepts used, as well as in their duration in the studies cited, we have thus chosen to use the umbrella term “digital deprivation” in this manuscript to refer to all types of intervention of different durations to be as exhaustive as possible. In addition to the concepts mentioned above, digitalization and digital deprivation can influence numerous dimensions linked to the psychophysiological state of individuals. They can also change how time is managed and perceived^53^, which should be considered in an increasingly fast-paced society where time is often precious. Advances in research into the effects of digitalization and digital deprivation are therefore examined in detail in the following sections.

### Time perception and digital technology

Time perception refers to how individuals experience the passage of time, which is subject to personal interpretation^54^. It involves the subjective experience and judgment of temporal intervals, including phenomena like simultaneity, successiveness, temporal order, and duration, influenced by cognitive processes and neural mechanisms^55^. Temporal estimations tend to differ widely from physical reality and might be easily modified^56^. Intrinsic factors, such as boredom, poor self-regulation, social media addiction risk and impulsivity, can lead to a slower perceived passage of time^36,57,58^. Temporary or psychological factors, such as depressive disorders, may also contribute to a feeling of time passing more slowly^59^. Contextual factors, such as the lockdown imposed during the COVID-19 pandemic resulted in a similar phenomenon^60^. During this period, higher levels of negative emotions, as well as less social satisfaction, were associated with a slower perceived passage of time^61,62^.

Using digital technologies also has an impact on time perception. Smartphone usage has been found to reduce the feeling of perceived boredom, especially when people are cognitively involved on their devices in hedonic (entertainment) or eudaemonic (information seeking, sociability) activities^63^. Several surveys and qualitative studies also suggested that smartphones, virtual calendars, or technological tools linked to the educational field might create a feeling of acceleration in tasks, of saving time for work and relaxation, of smart control of personal time, but also lead to more trouble with long-term thinking and planning^64–66^. Also, this sense of acceleration can give the impression of running out of time^67^. Furthermore, cognitive absorption may occur when using an application or software. It involves a state of total concentration, leading to an unawareness of the time that has passed^68^. This is particularly true for gamers who tend to lose time perception when playing video games^27^. This phenomenon of cognitive absorption could also predict addiction to social networks^69^. Individuals who experience low levels of boredom tend to underestimate time more, which might be the case when engaging in a digital activity^70^. More generally, the daily use of technology has fundamentally changed the perception and consumption of time. Individuals are continuously informed of the exact time, can access information and carry out tasks in real-time, and can schedule tasks flexibly to save time^53^. Thus, *organic* activities that do not involve digitalized instruments may be regarded as slow-paced^71^. As a result, an increasing number of individuals are turning to digital deprivation, intending to rediscover a more authentic and naturally paced lifestyle^72^.

However, the findings are ambivalent when the literature addresses the link between digital deprivation and time perception. It implies not using the Internet and the various digital tools available. This may be due to a deliberate rejection or lack of means to access them^73^. A few studies have examined the effects of short digital deprivation on time perception. People deprived of their smartphones can experience a feeling of boredom^74^. In a short time frame (several minutes), time might pass more slowly when watching an academic lecture compared to a video on social media use, but not when waiting without distraction^75^. Smartphone usage time, boredom proneness, intelligence, and working memory might also affect time perception^75^. Among young adults deprived of their smartphones for 48 h, opinions differed. Some felt that time passed more slowly, while others felt the opposite^76^. Over a longer period of one week, teenagers deprived of their phones had an ambivalent experience. They reported having the opportunity to spend more quality time with their loved ones, but they also had difficulty organizing themselves and carrying out their usual rituals and particularly felt that time passed much more slowly^77^. For a similar period but more focused on social media, a social media abstinence of one week led to an upward time distortion (i.e., time seems longer), in contrast to a control group that did not abstain. This temporal distortion after abstinence was particularly pronounced among users presenting a high risk of social media addiction^58^. Yet, the effects of digital deprivation are still relatively unclear, especially over a short time frame. Digitalization and its deprivation can also induce a change of state, such as anxiety or stress, and more generally have an impact on the psychophysiological state^12,21,23^. This is explored in the next section.

### Psychophysiological state

Changes in the psychophysiological state of individuals have been observed in various digital contexts on several physiological measures. Indicators such as heart rate, the standard deviation of intervals between normal R-peaks (SDNN), the root mean square of successive differences (RMSSD) of N-N intervals, and the power in low and high-frequency bands and its ratio (LF, HF, LF/HF) are often used to investigate changes in the physiological state of individuals^78,79^. Findings show rather negative effects of using digital devices on Heart Rate Variability (HRV)^80,81^. In the educational context, changes in HRV were found for students completing assignments online for three hours^80^. Changes were also found in young adults playing on a smartphone or watching someone playing a game (10 min each)^81^. However, it is not clear which indicators are the most sensitive to digitalization in both studies. Regarding electrodermal activity (EDA) indicators, it was shown that technostress increases skin conductance level (SCL), particularly among men^82^. Besides, highly anxious subjects show significantly lower skin conductance levels and amplitude responses than subjects with low anxiety^83^. As digital devices can induce anxiety, using them could have repercussions on their skin conductance measures. Performing a digital task can also require attentional resources and induce mental workload depending on the task demand^84^. Some physiological indicators such as HR or SCL have been proven to be reliable measures of cognitive workload which increases with task demand^85–88^. The frequency of non-specific skin conductance responses (NS-SCRs)^89^, a less frequently used indicator, is sensitive to physical activity^90^ but findings seem more contrasted for its sensitivity to changes in workload^91,92^. Short-term digitalization can also induce changes in the psychophysiological state. A good example are the notifications, now widespread on the digital devices we use daily, which trigger SCRs distinct from those triggered by arbitrary stimuli, whether auditory or vibrotactile^93^. Overall, fewer findings were made on the sensitivity of HRV indicators to digitalization than skin conductance.

The effects of digital deprivation on our psychophysiological state have also been investigated but results are disputed^21,74^. Complete deprivation of smartphone use or social media for a week showed positive effects such as a reduction of physiological stress measured through skin conductance^23^, less FOMO, and an improvement in mental wellbeing^26^. However, another study tested a reduction of 10 min per day on several social media platforms for three weeks and authors only found a reduction of depressive symptoms, but no significant changes in anxiety, FOMO, and wellbeing scores^94^. However, other studies found negative effects of digital deprivation^22,24,25^. With a similar deprivation duration of social media of one week as in^24,26^ rather found a decrease in students’ wellbeing and an increase of negative affect. Smartphone deprivation over a day did not show a significant effect on students’ mood and anxiety but increased craving^22^. Besides, the stress measured via saliva during a situation of social exclusion (smartphone use vs. smartphone presence vs. no smartphone) was higher when participants couldn’t access their smartphones but see them^25^. Similar results were found with mothers during breastfeeding as greater physiological stress was perceived when their smartphone was stored in their bag with silent mode than when the phone was stored in non-silent mode or used by the participants^95^. These studies also suggest that involuntary digital deprivation could create physiological stress in different contexts, especially if the phone is inaccessible. These findings also support that the presence of the smartphone (visual or audible), without being used, may already be sufficient to regulate the stress generated by digital deprivation.

These contrasted findings on the effect of digital deprivation on wellbeing were revealed by Radtke and colleagues in their meta-analysis^21^. They explain this by differences in experimental designs (e.g., type and duration of digital deprivation), which suggests the need for new studies with different experimental designs. Although few or no studies are explicitly assessing together in one study digital deprivation, time perception, and psychophysiology, some report observable effects of time and boredom on psychophysiological signs, or conversely. According to^96^, boredom is associated with higher arousal as measured with skin response and heart rate. Inversely, higher levels of SCR (caused by sounds with negative emotional valence) generate longer subjective durations^97^. However, the literature integrating these different fields needs more evidence.

## The current study

According to the above literature review, no study has examined the effect of short-term deprivation (less than a day) on both time perception and psychophysiological state. Contradictory results were found regarding the impact of digital deprivation on psychophysiological measures^21^ and no study investigated its effect on HRV measures. This study aimed to fill this research gap by assessing the effect of short digital deprivation on the psychophysiological state and time perception. It was tested through an artificial waiting period (7 min 30 s) experienced by a healthy population in a laboratory. To this end, participants were randomly assigned to one of three experimental conditions: free use of the smartphone (DA for Digitally Active), digital deprivation but performing a non-digital task (DDA for Digitally Deprived but Active), or digital deprivation and not performing any task (DDP for Digitally Deprived and Passive). Creating these three experimental groups enabled us to provide a finer insight into the real impact of the digital nature of a task and distinguish between situations of passive and active digital deprivation. For this study, the following hypotheses were formulated based on the literature review:

- (H1) Digital deprivation will have a significant effect on time perception.
  - (H1a) Participants in a passive digital deprivation condition (DDP) will be more bored than participants engaged in a task, whether digital or not (DA or DDA), due to the cognitive absorption caused by both tasks^30,63,68,74^.
  - (H1b) Consequently, participants in a passive digital deprivation condition (DDP) will feel that time passed more slowly, think more often about time, and show an upward time distortion (i.e., more time passed), contrary to participants engaged in a task, whether digital or not (DA or DDA)^30,58,63,70^. Our hypothesis slightly differs from results found by^75^ as the chosen non-digital task (i.e., sudoku) might be more engaging and demanding for the participants than watching an academic lecture.
- (H2) Digital deprivation will have a significant effect on the physiological state.
  - (H2a) The mean tonic EDA level of participants in a digital deprivation condition (DDP and DDA) will be lower than for participants engaged in a digital task (DA). This should be even more pronounced during passive deprivation (DDP)^23,85,87^. However, no hypothesis can be formulated for the effect to be found on the frequency of NS-SCRs as contradictory results were found in the literature^91,92^.
  - (H2b) Participants’ HRV in a digital deprivation condition (DDP and DDA) will be lower than for participants engaged in a digital task (DA). This should be even more pronounced during passive deprivation (DDP)^80,81,85,87^.
- (H3) Digital deprivation has a significant effect on the self-reported affective state.
  - (H3a) Self-reported participants’ arousal in a digital deprivation condition (DDP and DDA) will be lower than for participants engaged in a digital task (DA).
  - (H3b) Participants in a passive digital deprivation condition (DDP) will report a higher increase of negative affect due to boredom than participants engaged in a task (DDA or DA)^24^.

## Materials and methods

### Participants and experimental design

90 young participants (M = 21.73, SD = 1.98) including 76 women were enrolled in this study. They were mostly students at the University of Fribourg (Switzerland). Informed consent was obtained from all participants. The research was approved by the internal ethical review board of the University of Fribourg (Reference number: 2023–877). The research was performed in accordance with the local and national regulations and guidelines, and in accordance with the Declaration of Helsinki. The presented experiment was part of a larger study consisting of 3 experimental phases (baseline, waiting period, reward task). The current manuscript focuses only on the baseline and waiting period for better clarity. The experimental phase (baseline vs. waiting period vs. reward task) was a within-subject factor, while the experimental condition during the waiting period was a between-subject factor:

- Digitally Active (DA): Participants were prompted to use their smartphones freely.
- Digitally Deprived but Active (DDA): Participants were prompted to complete a sudoku on paper while waiting.
- Digitally Deprived and Passive (DDP): Participants were prompted to wait without performing any task while waiting.

Participants were randomly assigned to the three experimental conditions (30 subjects per condition). Table 1 shows the main socio-demographic information on participants, overall and depending on their experimental condition during the waiting period. The study was carried out by three experimenters who passed participants from randomized conditions.

**Table 1:** Socio-demographic information on participants and ratings from the French versions of the problematic internet use questionnaire (PIUQ-9), the depression anxiety stress scales (DASS-21), and the Barratt impulsiveness scale (BIS-11), overall and depending on their experimental condition during the waiting period.

	DA (*n* = 30)	DDA (*n* = 30)	DDP (*n* = 30)	Total (*n* = 90)	Reliability Indicator
	M (SD) or Frequency (%)	M (SD) or Frequency (%)	M (SD) or Frequency (%)	M (SD) or Frequency (%)	Cronbach’s Alpha (α)
Gender (female)	25 (83.33%)	27 (90%)	24 (80%)	76 (84.44%)	*-*
Laterality (right)	28 (93.33%)	26 (86.67%)	27 (90%)	83 (92.22%)	*-*
Professional situation (student)	28 (93.33%)	29 (96.67%)	29 (96.67%)	86 (95.56%)	*-*
Age (years)	21.70 (2.33)	21.72 (2.23)	21.73 (1.56)	21.73 (1.98)	*-*
PIUQ-9	23.2 (4.82)	21.97 (6.18)	20.10 (5.35)	21.76 (5.57)	0.799
DASS-21 (Depression)	11.73 (9.92)	8.80 (7.87)	8.07 (7.29)	9.53 (8.49)	0.890
DASS-21 (Anxiety)	13.13 (11.05)	10.13 (8.69)	10.93 (7.06)	11.40 (9.07)	0.823
DASS-21 (Stress)	15.73 (9.44)	18.67 (7.92)	15.93 (8.30)	16.78 (8.58)	0.821
BIS-11 (Impulsiveness)	48.70 (5.90)	46.70 (6.94)	46.60 (7.68)	47.33 (6.87)	

Note: DA = Digital Active, DDA = Digitally Deprived Active, DDP = Digitally Deprived Passive.

### Material and instruments

The participant was in a separate room, seated in front of a screen (Dell, 15,6”), with a keyboard and a mouse to answer questionnaires and perform the reward task. The screen displayed a copy of the laptop screen located in the experimenter’s room, with the time hidden. Another computer in the experimenter room was used to configure the physiological sensors and launch the video recording. Participants were equipped with two ambulatory physiological sensors (Movisens). A first sensor (EDAMove4, 32Hz) measuring the EDA was plugged into a wristband worn on the non-dominant hand, and connected to two Ag/AgCl electrodes placed on the outer part of the palm. A second sensor (ECGMove4, 1024 Hz) collecting the participants’ electrocardiogram (ECG) was plugged into a chest strap attached to the sternum location, which recorded the participant’s ECG through dry electrodes. During the waiting period, participants in the DA condition had to use their smartphones. Participants in the DDA condition were asked to fill grids of sudoku on paper (easy difficulty, EasyBrain, 2022).

### Measures

Various measures were computed from the raw EDA and ECG signals collected throughout the experiment. HRV measures such as HR, RMSSD, LF/HF were computed^78,80,85–88,98^. The mean tonic level and the number of non-specific skin conductance responses (NS-SCRs) per minute (also called frequency) were selected as EDA indicators^23,86,87,89,91^. A glossary explaining the abbreviations used, as well as the meaning of physiological signals and measures, can be found in Table 2 at the end of the manuscript.

**Table 2:** Glossary explaining the abbreviations used, as well as the meaning of physiological signals and indicators.

Indicator	Units	Description	Physiological significance
EDA mean tonic or SCL	Hz	Mean level of tonic electrodermal activity (EDA), also called Skin Conductance Level (SCL)	Sympathetic indicator May increase when sympathetic activity increases
NS-SCRs	Hz	Frequency of non-specific skin conductance responses (NS-SCRs)	Sympathetic indicator May increase when sympathetic activity increases
HRV	/	Heart Rate Variability	
HR	Bpm	Mean heart rate	Indicator sensitive to sympatho-vagal changes May decrease with increased parasympathetic activity
RMSSD	ms	Root mean square of successive difference between heartbeats	Mainly parasympathetic indicator
LF	ms^2^	Low frequency (LF) power of heart rate variability	Indicator sensitive to the orthosympathetic system and vagal influences 0.04–0.15 Hz
HF	ms^2^	High frequency (HF) power of heart rate variability	Indicator of parasympathetic activity 0.15–0.4 Hz
LF/HF ratio	/	Ratio between low and high frequency poser of heart rate variability	Index reflecting sympatho-vagal balance

Note: Hz = Hertz, bpm = beats per minute, ms = milliseconds.

Before the baseline phase, participants had to fill in a first questionnaire assessing their socio-demographic profile (current professional situation, highest degree, age), their impulsiveness measured with the French validated version of the Barratt Impulsiveness Scale (BIS-11, 22 items)^99^, their use of the internet during the last 6 months assessed with the validated French version of the Problematic Internet Use Questionnaire (PIUQ-9)^100^, and their depression, anxiety, and stress level measured with the French version of the Depression Anxiety Stress Scales (DASS-21)^101–104^. Table 1 shows participants’ ratings for BIS-11, PIUQ-9 and DASS-21.

After the waiting period, participants in the DA condition were asked if they used a digital device (“Did you use a digital device (smartphone, computer, tablet, connected watch, etc.) during the waiting phase?“; Yes/No), what type of digital tools they used and activities they engaged in (“What digital tools did you use during the waiting phase? What activity(ies) did you carry out during the waiting phase? On which application(s) or software(s)? Give as much detail as possible in your answer.“; Long textual answer), and if they looked at the time on this digital tool (“Did you look at the time during the waiting phase?“; Yes/No).

Time perception was assessed two times (after the waiting period and after the reward task), with four questions adapted from ^36^ and ^57^ and translated into French. Participants had first to report intuitively the duration of each period (TimeDuration: “Intuitively, without thinking about it, the *experimental phase* lasted X minutes and X seconds.“). This variable was converted to seconds for analysis. Participants also had to self-report how often they thought about time (TimeFrequency: “How often did you think about time during the *experimental phase*?”; 0 = Not at all – 1000 = extremely often), the speed at which time passed (TimeSpeed: “How fast did time pass for you during the *experimental phase*?”; 0 = Extremely slowly – 1000 = extremely fast), and to which extent they were bored (Boredom: “How bored were you during *experimental phase*?“; 0 = Not bored at all − 1000 = extremely bored) on 100-mm visual analog scales (VAS).

The participants’ affective state was assessed after each experimental phase using the Self Assessment Manikin (SAM)^105^. The correlations between the computerized version of the SAM valence and arousal scales and their semantic differential scores are respectively 0.97 and 0.94. Thus, two 5-point pictorial scales assessing valence and arousal were created using the pictograms from the original publication (1 = low valence/arousal, 5 = high valence/arousal). Additional questions were added on top of the pictorial scale (Valence: “To what extent did you feel positive or negative during the *experimental phase*?“; Arousal: “To what extent did you feel stimulated or calm did you feel during the *experimental phase*?“). Results on impulsiveness, depression, stress and anxiety, and the reward task are not presented in this manuscript.

### Experimental procedure

Participants were welcomed to the lab and all had to remove their bracelets, jewelry, and watches. Participants in the DA condition were asked to leave their smartphones in a box next to them on the table. They could leave their belongings near them in the participants’ room. Participants in the DDA and DDP conditions were asked to leave their smartphones in a box with their belongings in the experimenter’s room (i.e., no view on their smartphone). A cover story was used to explain the purpose of the experiment and not to mention time perception. Participants were told that they were participating in a study investigating the effect of digitalization related to reward mechanisms on the psychophysiological state of the population. They were also told they would win the amount of money gained in the reward task. The cover story was revealed at the end of the experiment.

After signing the consent form, physiological sensors were attached to the participants who filled in the first questionnaire. The baseline phase lasted five minutes to collect physiological signals at rest. Participants were explicitly told that it lasted five minutes and that they should stay still and breathe normally. At the beginning and end of each phase, participants had to tap twice on the EDA sensor to add a timestamp and confirm orally to the experimenter that the sensor vibrated. The experimenter came back to his/her room and started the video recording (used to control the engagement in the digital task afterward).

At the end of the baseline, participants assessed their valence and arousal. Meanwhile, the experimenter simulated a bug with the physiological recording (i.e., the timestamp did not work), and asked the participant to tap again. The latter marked the beginning of the waiting phase which lasted 7 min and 30 s^36,57^. The experimenter confirmed the “bug” and gave instructions to participants depending on their condition. Participants in the DA condition were prompted to use their smartphones and perform any activity they wanted. Those in the DDP condition were asked to wait without doing any activity (i.e., digitally deprived). Those in the DDA condition were asked to perform a non-digital task. An easy sudoku was chosen because it required participants to engage in a motor activity with their hands similar to the use of a the smartphone, while having low cognitive engagement given the difficulty of the task. The experimenters simulated mouse clicks, keyboard tapping, and sighs to make the bug credible. Participants could hear but not see what was happening next door in the experimenter’s room. At the end of the waiting phase, participants evaluated their time perception, valence, and arousal during the waiting phase.

After that, the reward task started. All the instructions were written on the screen, and participants were reminded that they would win the money. At the end of the task, they assessed again their time perception, valence, and arousal during the task. Physiological sensors were removed. The experimenter asked them for general feedback on the experiment, to understand if they believed the cover story, which was then revealed. The money won in the reward task was given in cash, and participants were thanked.

### Statistical analysis

For the manipulation check, a comparison of the scores of problematic use of Internet (PIUQ-9), stress, anxiety, depression (DASS-21), and impulsiveness (BIS-11) between the groups at baseline was carried out. None of these measures respected the normality so Kruskal-Wallis H tests were conducted, except for the stress score from the DASS-21 for which an ANOVA was carried out.

Five participants in the DA condition were excluded from the analysis because they used their smartphones for less than half the waiting period (3 min and 45 s). Besides, one participant in the DA condition was excluded from the analysis because he/she read a book for most of the waiting period.

After removing participants who did not meet the criteria for the DA condition, aberrant values were excluded using the quartiles (Q) and the interquartile range (IQR) when they were higher than Q3 + 3*IQR, or lower than Q1–3*IQR. Table 3 shows the number of participants removed from the analysis of each dependent variable, when the value was above or below the exclusion threshold.

**Table 3:** Number of participants removed from the statistical analysis of each dependent variable due to aberrant values. *N is the number of participants after removing participants who did not Meet the criteria of digital engagement and before removing outliers.*

	Participants excluded		
Measure	DA	DDA	DDP
	*N* = 24	*N* = 30	*N* = 30
TimeDuration	1	2	0
TimeFrequency	0	0	0
TimeSpeed	0	0	0
Boredom	0	0	0
HR	0	0	0
RMSSD	1	0	2
LF/HF	1	0	1
Tonic EDA	0	1	2
Freq. of NS-SCRs	3	1	1
Valence	0	0	0
Arousal	0	0	0

Note: DA = Digital Active, DDA = Digitally Deprived Active, DDP = Digitally Deprived Passive.

The normality of data distribution for each condition was then checked using the Shapiro-Wilk test and Q-Q plots. If the normality and homogeneity of variances were respected, a one-way or factorial ANCOVA was carried out to investigate the effect of the experimental phase (i.e., time), experimental condition, and interaction effect on the dependent variable, with the gender as covariate. Post-hoc tests with Bonferroni correction were carried out when a significant difference was found. If the normality was not respected, transformation techniques were tested. Otherwise, non-parametric tests were selected.

Regarding time perception measures, only the TimeSpeed measure followed a normal distribution without transformation. Levene’s test showed that the homogeneity of variances was respected (F = 0.17, *p* = .84) so a one-way ANCOVA with the experimental condition as between-subjects factor and gender as covariate was carried out on this measure. Even transformed (sqrt, log10, 1/x), the other time perception measures (TimeDuration, TimeFrequency, and Boredom) did not follow normal distribution. It was thus chosen to run non-parametric Kruskal-Wallis H tests, with experimental condition and gender as factors. Dunn’s post-hoc tests with Bonferroni were done when the results were statistically significant.

Regarding physiological measures, the EDA tonic mean level and the frequency of NS-SCRs were transformed in log(1 + x) to follow a normal distribution. HR followed a normal distribution without transformation. Both RMSSD and LF/HF were log-transformed. The homogeneity of variance was respected so a factorial ANOVA was run separately for each physiological measure, with gender as covariate.

Regarding affective state, valence and arousal measures (5-point Likert-scales) were far from normality. A Friedman test was run with the time measurement (after baseline vs. after waiting period) included as a within-subject factor. Wilcoxon signed-rank tests were also carried out to investigate further the differences between the baseline and the waiting period for each experimental condition and gender. Besides, Kruskal-Wallis tests were done to compare the participants’ ratings depending on their experimental condition and gender, at baseline and during the waiting period.

## Results

### Descriptive statistics and manipulation check

A comparison of the problematic use of internet (PIUQ-9: H(2) = 4.68, *p* = .10), impulsiveness (BIS-11: H(2) = 2.73, *p* = .25), depression (H(2) = 2.76, *p* = .25), stress (F(2, 87) = 1.10, p *=* .34, *η*_*p*_2 = 0.02, *p* = .22), and anxiety (H(2) = 0.94, *p* = .62) scores among all the participants of each experimental group did not reveal any significant differences between the groups at baseline (see Table 1 for descriptive statistics).

Table 4 reports the descriptive statistics for each dependent variable and experimental phase split by experimental condition, after excluding extreme values. Among those considered in the analysis, 10 participants in the DA condition reported they watched the time on their smartphone during the waiting period. They mostly sent or answered messages on smartphones.

**Table 4:** Descriptive statistics for each dependent variable and experimental phase (baseline and waiting period), split by experimental condition (DA = Digitally Active, DDA = digitally deprived but active, DDP = digitally deprived and passive), after excluding outliers. Table 2 explains the abbreviations used and Table 3 shows the number of participants considered in these statistics.

Measure	Baseline			Waiting Period		
	DA	DDA	DDP	DA	DDA	DDP
TimeDuration	NA	NA	NA	368 (110)	302 (70)	364 (106)
TimeFrequency	NA	NA	NA	299 (287)	271 (299)	423 (295)
TimeSpeed	NA	NA	NA	590 (199)	645 (183)	456 (184)
Boredom	NA	NA	NA	192 (172)	169 (231)	527 (273)
HR (beats per minute)	79.68 (9.80)	76.56 (9.61)	78.00 (13.22)	78.70 (11.03)	79.02 (10.06)	77.51 (12.64)
RMSSD	3.58 (0.49)	3.91 (0.47)	3.67 (0.53)	3.58 (0.48)	3.67 (0.48)	3.71 (0.48)
LF/HF	0.38 (0.83)	0.38 (0.88)	0.46 (0.72)	0.63 (0.79)	0.50 (0.69)	0.43 (0.64)
Tonic EDA	1.55 (0.60)	1.58 (0.58)	1.51 (0.61)	1.60 (0.58)	1.65 (0.59)	1.78 (0.57)
Freq. of NS-SCRs	1.53 (0.66)	1.57 (0.41)	1.61 (0.50)	1.59 (0.50)	1.60 (0.49)	1.59 (0.50)
Valence	3.50 (0.66)	3.53 (0.78)	3.67 (0.66)	3.67 (0.64)	4.03 (0.85)	3.50 (0.63)
Arousal	1.79 (0.78)	1.97 (0.89)	2.40 (0.93)	2.08 (0.93)	2.56 (1.13)	2.23 (0.77)

(17) or browsed social media (8). Other activities carried out were browsing online for searching opening times of a store (1), reading the news (1), sending emails (1), studying (1), doing online sudoku (1), playing (1), and planning a task (1). The most used applications were social media platforms such as Instagram (13), WhatsApp (12), Snapchat (7), Twitter (1), and Facebook (1). Other applications were a web browser (1), a news application (1), Discord (1), iMessage (1), Outlook (1), Goodnotes (1), and Candy Crush (1).

### Time perception

**Fig. 1:**
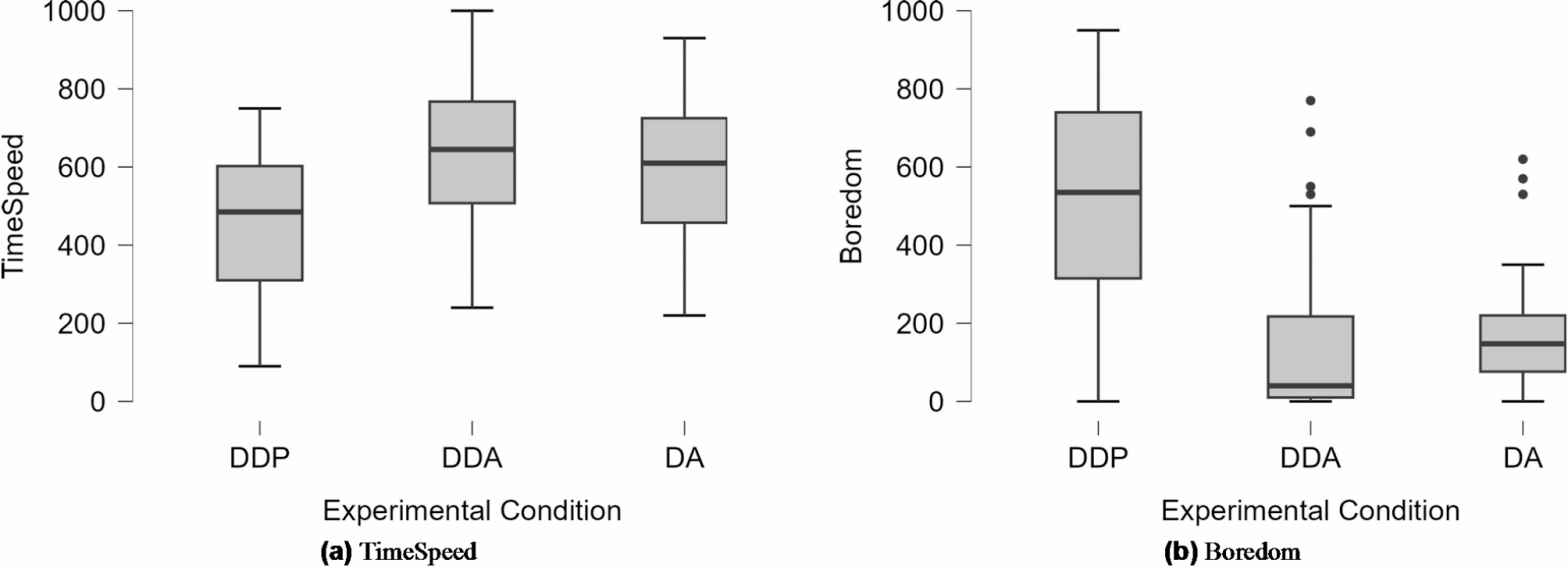
Significant effect of experimental condition (digitally active, DA; digitally deprived but active, DDA; digitally deprived and passive, DDP) on speed of passage of time (TimeSpeed) and boredom (Boredom).

There was a marginal significant effect of the experimental condition on the estimated duration of the waiting period (TimeDuration) reported by participants (H(2) = 5.83, *p* = .054). Post-hoc comparisons showed no significant differences between conditions. There was no significant effect of gender on TimeDuration (H(1) = 1.53, *p* = .22). Regardless of their condition, the participants underestimated the duration of the waiting period (M = 343.73 s, SD = 100.15 s). A significant effect of the experimental condition was found on the speed of passage of time (TimeSpeed; F(2, 81) = 7.68, p *<* .001, *η*2 = 0.16). This is shown in Fig. 1a. Post-hoc tests revealed a significant difference between participants in the DDP condition who felt that the time passed slower than participants in the DDA (*p* < .001) or DA (*p* = .03) conditions. However, the speed of passage of time did not differ for participants in the DDA and DA conditions (*p* = .87). Also, a significant effect of the experimental condition was found on boredom during the waiting period (H(2) = 26.06, p *<* .001). It is shown in Fig. 1b. Post-hoc comparisons showed that participants in the DDP condition felt significantly more bored than participants in the DDA (p *<* .001) and DA (p *<* .001) conditions. There was no significant difference between the DA and DDA groups (*p* = 1.00). There was no effect of gender as covariate on TimeSpeed (F(1, 81) = 0.14, *p* = .70, *η*2 = 0.00) and on boredom (H(1) = 0.41, *p* = .52). Finally, no effect of experimental condition (H(2) = 4.94, *p* = .08) and gender (H(1) = 1.31, *p* = .25) were found on the frequency at which participants thought about the time (TimeFrequency) during the waiting period.

### Physiological state

A significant effect of the experimental phase was found on EDA tonic mean level (F (1, 76) = 7.77, p *<* .01, *η*_*p*_2 = 0.10). The mean tonic EDA level of participants was higher during the waiting period (M = 1.70, SD = 0.59) compared to the baseline period (M = 1.56, SD = 0.60), regardless of their experimental condition. Otherwise, there was no significant effect of the experimental condition or gender alone (Fs < 1), no significant interaction effect between the experimental phase and gender (F *<* 1), and no significant interaction effect between experimental phase and condition (F(2, 76) = 2.27, p *=* .011, *η*_*p*_2 = 0.06). Also, there was no significant effect of the experimental phase, experimental condition, gender, or interaction effect on the frequency of NS-SCRs (Fs *<* 1).

**Fig. 2:**
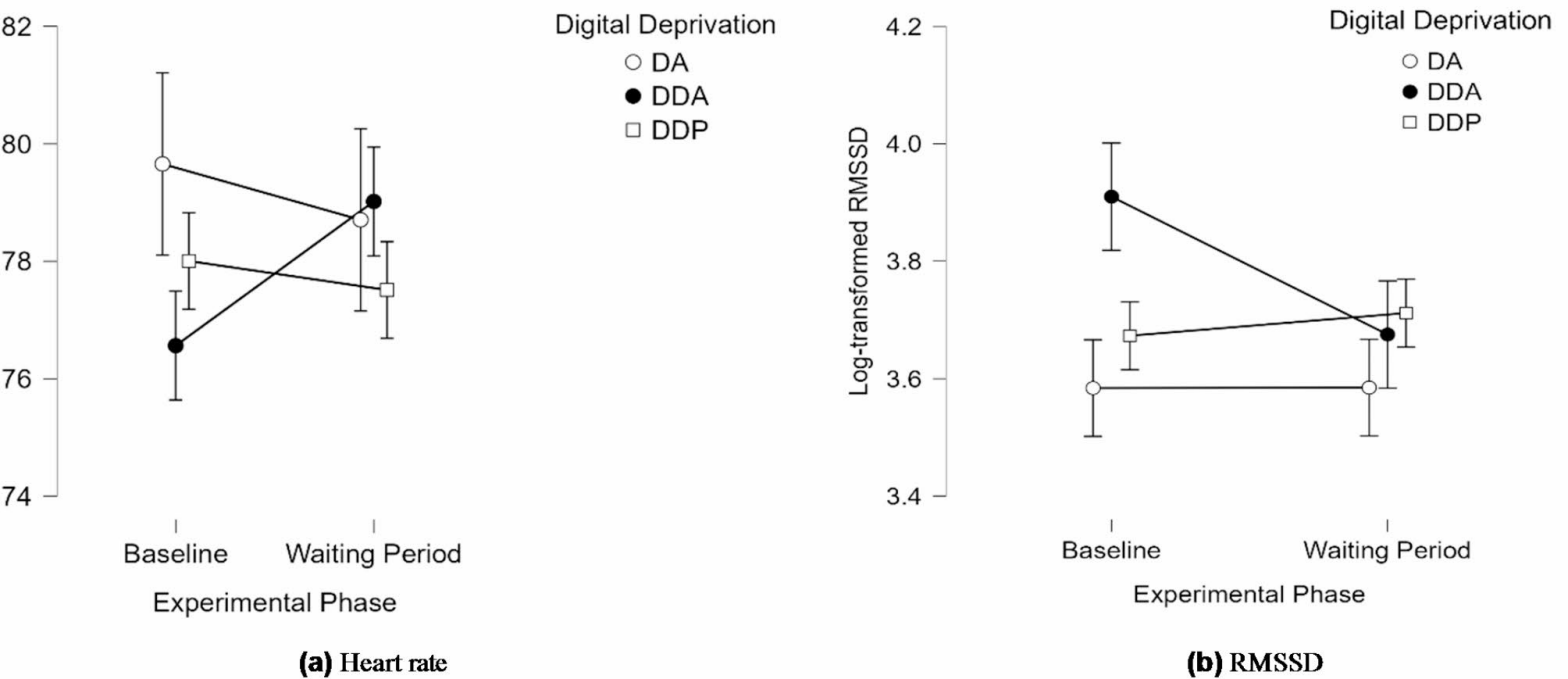
Interaction effect of experimental condition (digitally active, DA; digitally deprived but active, DDA; digitally deprived and passive, DDP) and experimental phase (baseline, waiting period) on the participants’ heart rate and RMSSD. Error bars represent confidence intervals at 95%.

Regarding HRV measures, a significant interaction effect between the experimental phase and condition was found on HR (F(2, 77) = 6.23, p *<* .01, *η*_*p*_^*2*^ = 0.14), which can be seen in Fig. 2a. Post-hoc tests averaged with levels of gender did not reveal significant difference between the baseline and the waiting period for any experimental condition (DDA: t(77) = - 2.82, *p* = .09, d = - 0.22; DA: t(77) = 1.12, *p* = 1.00, d = 0.09; DDP: t(77) = 0.69, *p* = 1.00, d = 0.05), but suggest an increase for the DDA condition. No significant effect of gender alone (F(1, 77) = 3.55, *p* = .06, *η*_*p*_2 = 0.04) and interaction effect with the experimental phase (F < 1) were found on HR. No significant effect of the experimental condition or the experimental phase alone were found on HR (Fs *<* 1).

A significant interaction effect between the experimental phase and experimental condition (F(2, 74) = 8.34, p *<* .001, *η*_*p*_2 = 0.18) was found on the log-transformed RMSSD. The interaction effect can be seen in Fig. 2b. Post-hoc tests revealed that RMSSD decreased from baseline to the waiting period for participants in the DDA group (t(74) = 4.41, p *<* .001, d = 0.49), but while it remained stable for participants in other conditions (DA: t(74) = 0.47, *p* = 1.00, d = 0.06; DDP: t(74) = - 0.3, *p* = 1.00, d = - 0.00). No significant effect of the experimental condition (F(2, 74) = 1.26, *p* = .29, *η*_*p*_2 = 0.03), of the experimental phase (F(1, 74) = 2.15, *p* = .15, *η*_*p*_2 = 0.03), of gender (F < 1), or of the interaction effect between experimental phase and gender (F(1, 74) = 1.31, *p* = .26, *η*_*p*_2 = 0.02) was found.

On log-transformed LF/HF measures, no significant effect of the experimental phase (F(1, 75) = 1.56, *p* = .22, *η*_*p*_2 = 0.02), of the experimental condition (F < 1), of the gender (F(1, 75) = 1.31, *p* = .26, *η*_*p*_2 = 0.02), of the interaction between experimental phase and condition(F(2, 75) = 1.03, *p* = .36, *η*_*p*_2 = 0.03), and of the interaction between experimental phase and gender (F < 1) were found.

### Affective state

**Fig. 3:**
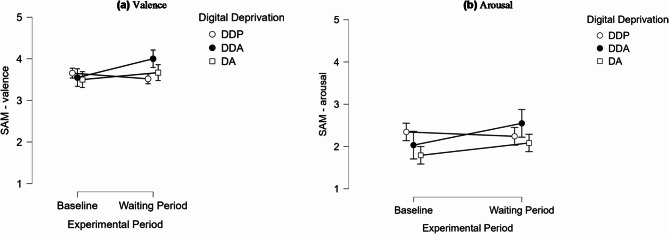
Interaction effect of experimental condition (digitally deprived and passive, DDP; digitally deprived but active, DDA; digitally active, DA) and experimental phase (baseline, waiting period) on the participants’ valence and arousal. Error bars represent confidence intervals at 95%.

The Friedman test revealed a significant effect of experimental phase on valence (X^2^(1) = 4.80, p *<* .05) and a marginally significant effect on arousal (X^2^(1) = 3.79, *p* = .05). Regardless their experimental condition or gender, participants reported a more positive valence (M = 3.74, SD = 0.75) and a higher arousal (M = 2.31, SD = 0.97) during the waiting period than during the baseline (Valence: M = 3.57, SD = 0.70; Arousal: M = 2.07, SD = 0.90). This can be seen in Fig. 3a and b.

To further explore the differences between the experimental groups, the Wilcoxon signed-rank tests revealed a significant difference in valence (W = 14.00, z = -2.79, p *<* .01) and arousal (W = 36.00, z = -2.37, p *<* .05) for participants in the DDA condition. Both increased from the baseline to the waiting period, as shown in Fig. 3a and b. However, no significant difference in valence and arousal was found for participants in the DDP (Valence: W = 24.00, z = 1.69, *p* = .07; Arousal: W = 52.00, z = 1.02, *p* = .30) and DA (Valence: W = 7.00, z = -1.18, *p* = .24; Arousal: W = 3.00, z = -1.86, *p* = .06) conditions between the baseline and the waiting period. The females’ valence was different between periods, but it was not the case for males (Females: W = 87.50, z = -2.23, *p* < .05; Males: W = 5.00, z = 0.00, *p* = 1.00). Arousal did not vary, regardless the gender of participants (Females: W = 171.50, z = -1.73, *p* = .07; Males: W = 6.00, z = − 0.94, *p* = .37).

Kruskal-Wallis tests showed a significant difference between the experimental conditions at baseline for the arousal (H(2)= 6.69, p *<* .05) but not for valence (H(2) = 1.32, *p* = .52). Dunn’s post-hoc tests with Bonferonni corrections showed that participants in the DA condition felt less aroused at baseline than participants in the DDP condition (*p* = .04). No significant differences were found between DDA and other conditions (DDP: *p* = .19; DA: *p* = 1.00). A significant effect of experimental condition was found on valence (H(2) = 7.28, p *<* .05) but not on arousal (H(2) = 2.69, *p* = .26) during the waiting period. Participants in the DDA condition felt more positive during the waiting period than participants in the DDP condition (p *<* .05). There was no difference in valence during the waiting period between DA and other conditions (DDP: *p* = .53; DA: *p* = .20). Besides, no effect of gender was found on valence and arousal both during baseline (Valence: H(1) = 0.49, *p* = .48; Arousal : H(1) = 0.47, *p* = .49) and waiting period (Valence: H(1) = 0.07, *p* = .78; Arousal : H(1) = 0.01, *p* = .94).

## Discussion

### Time perception

The first notable result was that participants underestimated the length of the waiting period, regardless of their experimental condition. This is known as downward time perception bias and is consistent with previous findings in the general population^58^. The large variance of the predicted duration of the waiting period suggests that elapsed time is a difficult concept to grasp and predict. Such variance in the perception of time between individuals has already been found in previous literature^36,106^. This is the case for all measures of time perception included in this study, particularly for the frequency with which people think about time (see Table 4). In addition, a significant effect of experimental condition was found on the speed of time passage and boredom during the waiting period. Passive digitally deprived participants (i.e. no task) felt that time passed more slowly and were more bored than participants engaged in a task, regardless of its digital nature. This finding is consistent with previous literature suggesting that subjective time generally slows down with boredom^57^. It is also consistent with another study suggesting that smartphone usage reduces the feeling of perceived boredom, especially when people are cognitively involved on their devices for entertainment, information seeking and social activities^63^. In this study, this was the case for participants engaged in a task, whether digital or not. These results suggest that the cognitive absorption induced by the task performed may have more impact on time perception than its digital nature. Cognitive absorption may have been sufficient in both task-engaged conditions to reduce boredom and thus lead to the same feeling about the speed of time passing^68,69^. Some avenues for further research on the effect of the digital nature of a task on time perception are proposed below.

In summary, H1a is confirmed, and H1b is only partially confirmed with regard to the slower passage of time for passive digitally deprived participants. Indeed, no effect of the experimental condition was found on the frequency with which participants thought about time during the waiting period. This suggests that cognitive engagement is linked more strongly to boredom and the speed with which time passes, but not so much to the frequency with which we think about time. Although, descriptively, participants in the waiting period thought more about time than in the other two conditions, suggesting that an effect would be achieved with more participants. This hypothesis should be further confirmed.

### Physiological state

Sudoku (easy level) was chosen because it involves motor actions with the hand similar to when interacting with the smartphone. It was assumed that it would require cognitive engagement and a mental load equivalent to smartphone use. However, the results of the HRV measurements suggest that the sudoku task was more cognitively engaging than the original design anticipated. Indeed, the participant’s heart rate increased (and thus RMSSD decreased) between baseline and the waiting period when conducting sudoku. However, this was not the case for participants using their smartphones or waiting. No effect was seen on LF/HF, but the waiting period may have been too short to observe significant changes in this measure. This measure was originally created for 24-hour recordings and correlates poorly with measures calculated from 5-minute recordings^79,107^. H2b is thus refuted. Furthermore, no effect of experimental condition was found on EDA measures, which refutes H2a. Task demand might have been different enough to differentiate the cognitive workload induced by the two tasks with heart rate and RMSSD, but not with skin conductance measures. In addition, using NS-SCR frequency was more exploratory in differentiating task-induced cognitive demand. The results align with^91^ rather than those found by^92^. If the non-significant effects found on the EDA measures and the significant ones found on the HRV are confirmed, this would mean that two tasks requiring a different cognitive load (Sudoku being more demanding than the free use of the smartphone) could have the same effect on the perception of time during the execution of this task (less boredom and time passing more quickly). In other words, the digital nature of the task, even if less demanding, would absorb as much and modify the perception of time in the same way as a non-digital but more demanding task. Another interpretation of these results is that because skin conductance also increased for participants in the DDP condition, we could not discriminate participants’ conditions with EDA measures but only with HRV measures. The fact that only an effect of time was found on EDA measures supports this interpretation. This suggests that different modalities of digital deprivation can have different impacts on EDA and HRV. This should be confirmed in future studies with both EDA and HRV measures included. Indeed, some physiological measures such as heart rate and blood pressure were found sensitive when being unable to answer to their ringing phone while engaging in a cognitive task^28^.

### Affective state

Regarding the self-reported affective state, the results suggest that the participants who did the sudoku reported a more positive valence and higher arousal during the waiting period than during the baseline, compared to other participants. This is coherent with results in our study found on HRV measures (i.e., the higher cognitive workload that it induced). Some participants also reported that they enjoyed doing the sudoku in their feedback. H3a and H3b are therefore refuted as the difference was found in the DDA condition compared to other conditions. We could have expected that the free use of a smartphone by participants (i.e., DA condition) to induce a more positive affect. As suggested in previous literature, we could also have observed a higher increase in negative affect for passive digitally deprived participants, which could be explained by boredom, higher anxiety levels or FOMO^27,63^.

### Limitations and avenues for further research

The sample of participants consisted predominantly of young females. It might not be representative of the global population, as age and gender can affect time perception and physiological state^79,108^. In terms of gender, participants were equally balanced across experimental groups but not overall (more women than men, see Table 1). The statistical analysis revealed no significant influence on all dependent variables except valence. With regard to participants’ age, given the very low standard deviation, it was impossible to investigate its effect in this study. The results obtained and discussed above are therefore valid for a young population. The study should be reproduced with participants of more varied ages, possibly separated by age group (young vs. old adults). Although participants primarily used their non-dominant hand with minimal movement, the physiological sensors and equipment may have introduced noise, potentially skewing the data and affecting the calculated indicators. Six participants had to be removed only in the DA group before the analysis because they did not engage (long enough) in a digital activity, which might have slightly skewed the reliability of results. Besides, three experimenters were involved in data collection. Despite their training to deliver consistent instructions and adhere to a standardized instruction sheet, slight variations in delivery may have occurred. Some participants might also have expressed skepticism towards the cover story involving the bug, which could have diminished the effects of digital deprivation. Nevertheless, most of them admitted they believed in it when giving their general feedback after the experiment. In the experimental conditions of digital deprivation, participants couldn’t see the smartphone. The results might have been different if they would have been able to see it but not use it^25,95^. As previously stated, it can be argued that the selected non-digital task had low ecological validity. However, digitally active participants were prompted to freely use the smartphone for better ecological validity. In modern society, individuals frequently use digital devices for short periods, using different applications according to individual preferences. Finally, conducting the study in a laboratory setting may have been intimidating for participants and does not fully capture real-world conditions. Implementing a similar study in a natural environment with longer and varied periods of digital deprivation would be of interest.

To further explore the influence of cognitive engagement (digital vs. non-digital) on time perception, a future study could use the same sudoku task in both digital and non-digital versions. This would make it possible to study whether there is any form of “digital effect”. Another option would be to use a cognitively less engaging task such as reading newspapers (in paper or digital format), as happening in real waiting rooms, what might reduce the effect of cognitive engagement in the control condition and be more ecologically valid- It would in any case be necessary to control beforehand that the cognitive engagement and mental workload induced in both condition are similar.

## Conclusion

The potential benefits of digital deprivation have been investigated in the literature but are still disputed. Moreover, its effect on both our perception of time and our psychophysiological state is still poorly understood. This study investigated the impact of short digital deprivation (passive or not) on psychophysiological state and perception during a short waiting phase (7 min 30) artificially created in the laboratory. The results of the conducted study contribute to the ongoing discussion on the effect of digital deprivation vs. digital engagement on psychological functioning.

Participants underestimated the length of the waiting period, confirming results already shown in the general population. Passive digitally deprived participants during the waiting phase felt that time passed more slowly and were more bored than participants engaged in a task, regardless of its digital nature. Thus, being engaged or not in a task over a short period seems to affect the perception of time and boredom, more than the digital nature of the task.

Completing the sudoku task seemed more cognitively engaging and generated more positive affect than free smartphone use, which lead participants to mainly send and reply to messages or go on social networks. The study suggests that people might benefit from performing non-digital tasks during short waiting phases, as these activities can be more engaging and enjoyable than passive digital engagement. This can be a practical recommendation for individuals seeking to optimize their waiting time for better psychological outcomes.

Another finding that should be investigated in future research is that skin conductance could increase even when being passive during a short waiting phase, opposingly to heart rate variability. Another similar study with longer or repeated digital deprivation and with a different non-digital task to perform (e.g. reading the news) should be carried out to confirm the results obtained in this study.

## Data Availability

The datasets generated and/or analysed during the current study are available in the OSF repository, DOI: https://doi.org/10.17605/OSF.IO/PK4TS (2024).
